# Dual-ligand surface engineering of Ni-based nanostructures for efficient urea electrooxidation *via* Ni^3+^ activation and charge-transfer modulation

**DOI:** 10.1039/d6ra00837b

**Published:** 2026-03-25

**Authors:** Wang Yifei, Li Jiayin, Luo An, Jin Yanxian, Xu Wei, Chen Dan, Yu Hua, Worathat Thitikornpong, Yu Binbin

**Affiliations:** a School of Pharmaceutical and Chemical Engineering, Taizhou University Taizhou 318000 China yubb@tzu.edu.cn; b Zhejiang Baima Lake Laboratory Co., Ltd Hangzhou 310053 China; c Department of Food and Pharmaceutical Chemistry, Faculty of Pharmaceutical Sciences, Chulalongkorn University Bangkok 10330 Thailand; d Center of Excellence in Natural Products for Ageing and Chronic Diseases, Chulalongkorn University Bangkok 10330 Thailand

## Abstract

Nickel-based metallic nanomaterials represent highly promising electrocatalysts for the urea oxidation reaction (UOR), enabling the simultaneous benefits of efficient hydrogen production and wastewater treatment. However, their catalytic performance is constrained by slow interfacial charge transfer and insufficient exposure of active Ni^3+^ sites. Herein, we propose a dual-ligand surface modification strategy employing glutaric acid (Ga) and ferrocenecarboxylic acid (Fc) as co-modifiers alongside phthalic acid as the primary linker, simultaneously optimising the geometric structure and electronic state of the nickel-based catalyst. The optimally modified nickel-based catalyst exhibits a rich array of surface defect morphologies, and XPS analysis confirms that under this modification scheme, the material's specific surface area increases moderately (71.3 m^2^ g^−1^), with a marked enhancement in the Ni^3+^/Ni^2+^ ratio. These characteristics effectively accelerate the Ni^3+^/Ni^2+^ redox conversion and promote the formation of the key active intermediate NiOOH, thereby achieving a low onset potential (0.49 V *vs.* SCE), minimal Tafel slope (20.98 mV dec^−1^), and excellent electrochemical durability. Electrochemical impedance and comparative analyses further reveal that glutaric acid-induced surface structural disorder is the dominant factor enhancing interfacial charge transfer. This study presents a ligand-directed surface engineering approach to construct defect-rich nickel-based electrocatalysts with high intrinsic activity, providing a novel technological pathway for sustainable hydrogen production and the resource recovery of urea-containing wastewater.

## Abstract 2

Using glutaric acid yielded a Ni material rich in surface defects and electronically modulated properties. The Ni/Ga catalyst exhibits outstanding urea oxidation performance with low potential (0.49 V *vs.* SCE) at 10 mA cm^−2^ and high stability.

## Graphical abstract

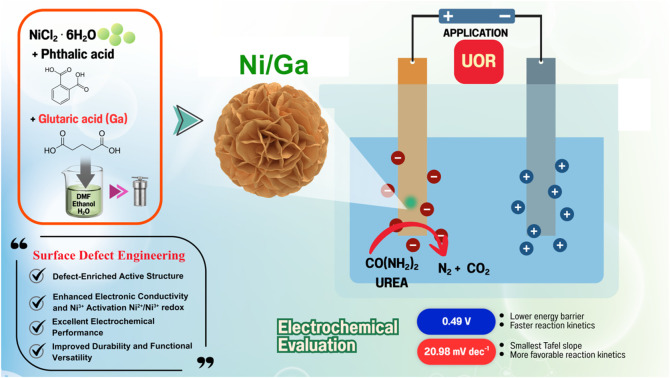

## 1. Introduction

The electrochemical oxidation of urea (UOR) has emerged as a promising alternative to the oxygen evolution reaction (OER) for sustainable hydrogen production, owing to its lower theoretical potential (0.37 V *vs.* 1.23 V for OER) and alongside the added benefit of simultaneous urea-rich wastewater remediation.^1–3^ Urea is abundant, non-flammable, and safe to handle, making it a suitable feedstock for direct urea fuel cells and alkaline electrolysis.^1–3^ The oxidation of urea releases nitrogen, carbon dioxide, and six electrons per molecule (CO(NH_2_)_2_ + 6OH^−^ → N_2_ + 5H_2_O + CO_2_ + 6e^−^), enabling energy-efficient hydrogen generation. However, this six-electron transfer process proceeds through multiple intermediates and exhibits slow reaction kinetics,^4–6^ necessitating the development of robust electrocatalysts to accelerate charge transfer and promote intermediate decomposition.

Among the diverse electrocatalysts explored for the UOR, nickel-based materials have received considerable attention owing to their earth-abundant composition, redox-active Ni^2+^/Ni^3+^ couples, and highly tunable porous structures.^7–9^ By introducing specific surface modifiers to enhance nickel's surface properties, researchers can effectively regulate the material's morphology and increase the exposure of active sites, thereby providing a straightforward and feasible approach to improving catalytic performance.^10,11^ However, nickel-based materials suffer from intrinsically low electrical conductivity, and limited exposure of active sites often hinders practical performance. Morphology control has proven critical for overcoming these limitations. For example, Li *et al.* reported that constructing hierarchical MOF@NiO/Ni nanorods enhanced stability and exposed more active sites, leading to superior urea electrolysis performance.^12^ Similarly, Cheng *et al.* demonstrated that reshaping Ni nanostructures from nanowires to nanoboxes significantly improved their electrocatalytic efficiency for the oxygen evolution reaction.^13^ Collectively, achieving precise structural evolution and performance optimisation through surface modification techniques is crucial for unlocking the potential of nickel-based materials in the UOR field.

Surface defect engineering serves as an effective strategy to enhance the performance of nickel-based electrocatalysts by introducing surface unsaturated site defects. This approach increases the density of exposed active sites and alters the local electronic environment.^11,12^ Concurrently, surface-functionalising agents offer a rational approach to modifying nickel surfaces, enabling regulation of the coordination geometry and oxidation state of metal centres. Introducing specific modifiers with steric hindrance or electron-inducing effects disrupts the long-range ordered arrangement on the metal surface, forming unsaturated metal sites with enhanced redox activity.^11^ Recent studies have validated these principles in practice. For instance, Qin *et al.* demonstrated that synergistic vacancy and doping engineering in Ni(OH)_2_ created unsaturated Ni coordination sites that significantly boosted UOR kinetics,^14^ while Gao *et al.* employed oxyanion engineering to suppress competing OER on Ni surfaces, achieving a current density of 323.4 mA cm^−2^ with 99.3% N-product selectivity.^15^ Nevertheless, these strategies have largely been applied in isolation, and the comparative effects of distinct functional modifiers, such as Ga and Fc, in simultaneously inducing Ni oxidation state evolution, defect formation, and UOR activity enhancement remain poorly understood.

In recent years, significant breakthroughs have been made in the development of nickel-based electrocatalysts for UOR. For example, Ge *et al.* employed a mild chemical modulation strategy to reconstruct the surface structure of a two-dimensional MOF, optimising its electronic properties and adsorption capacity, thereby effectively regulating the electronic structure of nickel centres.^16^ Additionally, Lou *et al.* adopted an electrochemical semi-sacrificial growth strategy to construct three-dimensional nanoplate arrays, which significantly enhanced both mass transfer and active site accessibility.^17^ Meanwhile, Xie *et al.* improved charge separation and transport efficiency during urea photoelectrolysis by constructing oxyhydroxide heterojunctions to modulate local charge distribution.^18^ Recently, Chen *et al.* constructed hierarchical porous Ni–WO_3_/NF nanosheet arrays with abundant heterointerfaces, enabling precise regulation of charge distribution at Ni sites; the heterointerfaces promoted Ni^3+^ generation and optimised affinity toward urea/CO_2_ intermediates, accelerating UOR kinetics.^19^ Notably, the catalyst achieved 200 mA cm^−2^ at 1.384 V *vs.* RHE with outstanding stability over 150 h. Along similar lines, Yi *et al.* demonstrated that a p–n Ni_3_S_2_/Co_3_O_4_ heterostructure on nickel foam exploited the built-in electric field at the heterointerface to promote urea adsorption and molecular decomposition, requiring only 1.288 V to reach 10 mA cm^−2^ with remarkable 100 h durability.^20^ You *et al.* further developed trifunctional Co_2_P/NiMoO_4_ heterostructures on nickel foam, simultaneously catalysing OER, HER, and UOR through synergistic bimetallic interface engineering.^21^ Beyond heterostructure approaches, emerging evidence highlights the pivotal role of ligand coordination in governing Ni active-site behaviour. Liu *et al.* directly imaged the stepwise *in situ* chemical transformation of a multidentate-ligand-capped Ni cubane nanocluster during urea electrolysis, revealing that labile ligand coordination sites serve as the catalytic active centres and that the hydrogen-bonding network of the ligand facilitates urea decomposition, providing direct molecular-level evidence that ligand identity critically governs UOR activity.^22^ Ajmal *et al.* further demonstrated that the nature of the coordinating ligand in Ni-based coordination compounds directly controls the electron-donation tendency of the Ni centre, controlling the ease of self-reconstruction into the active NiOOH phase and the resulting electrocatalytic performance.^23^ While these studies have provided critical insights into enhancing UOR kinetics through framework optimisation, heterostructure design, or heteroatom doping, the comparative roles of distinct non-bridging functional ligands in simultaneously tuning Ni^3+^ content, surface defect density, and charge-transfer efficiency for UOR remain largely unexplored.

This work departs from conventional bulk modification paradigms by introducing a ligand-mediated surface defect engineering strategy. Using Ga and Fc as non-bridging surface modifiers, we achieve controllable induction of surface lattice distortion and high-valent Ni^3+^ species. Unlike the aforementioned approaches focusing on complex framework engineering or doping, this study introduces a functional-ligand surface regulation strategy, employing Ga and Fc as surface modifiers to design nickel-based catalysts. The aim of incorporating these functional ligands is to induce surface structural defects and tailor the local coordination environment of Ni active sites, thereby boosting UOR performance. Three comparative materials, pure Ni, Fc-modified Ni (Ni/Fc), and Ga-modified Ni (Ni/Ga), were successfully synthesised *via* a one-step hydrothermal method and systematically characterised. Experimental results demonstrate that Ni/Ga exhibits the most outstanding UOR activity, with higher current density and optimised kinetic parameters. Through ligand-directed surface engineering, this work provides a rational and scalable route for designing defect-rich nickel-based electrocatalysts. Comprehensive analysis confirms that functional-ligand modification effectively increases the Ni^3+^ content, electrochemically active surface area, and charge-transfer efficiency, providing mechanistic insights for designing efficient catalysts in energy conversion and wastewater treatment.

## 2. Experimental section

### 2.1 Chemicals and reagents

Nickel(ii) chloride hexahydrate (NiCl_2_·6H_2_O), phthalic acid (C_8_H_6_O_4_), glutaric acid (C_5_H_8_O_4_), and ferrocenecarboxylic acid (C_11_H_10_FeO_2_) were purchased from Macklin Biochemical Co., Ltd (Shanghai, China). *N*,*N*-Dimethylformamide (DMF), ethanol (EtOH), potassium hydroxide (KOH), and Nafion solution (5% w/w) were obtained from Zhejiang Noah Chemical Technology Co., Ltd (Jinhua, China) and Shanghai Hesen Electric Co., Ltd (Shanghai, China), respectively. Urea (≥99.0%) and agar powder were supplied by Jiangsu Qiangsheng Functional Chemical Co., Ltd (Changshu, China) and Hangzhou Mumu Biotechnology Co., Ltd (Hangzhou, China), respectively. All chemicals were used as received without further purification. Ultrapure water (resistivity = 18.2 MΩ cm) was used in all experiments.

### 2.2 Synthesis of Ni

**Fig. 1:**
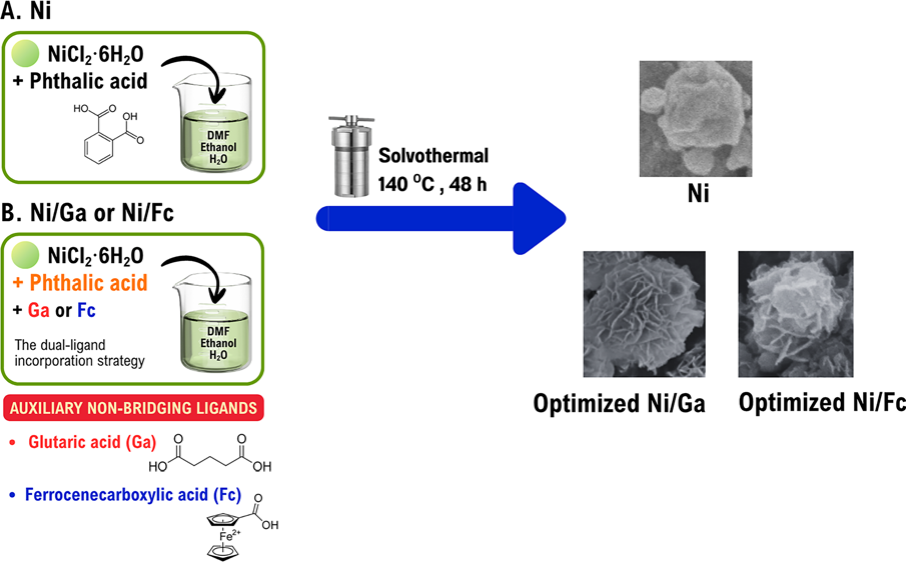
Schematic illustration of the synthesis routes for Ni-based materials. (A) One-step hydrothermal preparation of Ni material. (B) Synthesis of Ni/Ga and Ni/Fc using Ga or Fc as surface modifiers, respectively. The incorporation of Ga or Fc tailors the surface properties, leading to the formation of defect-rich structure.

The Ni was synthesised using a hydrothermal method. In a typical preparation, 64 mL of DMF, 4 mL of ethanol, and 4 mL of ultrapure water were mixed in a 100 mL Teflon-lined stainless-steel autoclave. Phthalic acid (250.45 mg, 1.5 mmol) was added, and the mixture was ultrasonicated until completely dissolved. Then, nickel(ii) chloride hexahydrate (356.54 mg, 1.5 mmol) was introduced under continuous stirring. The autoclave was sealed and heated at 140 °C for 48 h, and allowed to cool to room temperature. The resulting suspension was centrifuged at 8000 rpm for 5 min, and the obtained precipitate was washed three times with ethanol and dried under ambient conditions. A schematic representation of the synthetic procedure is shown in Fig. 1.

### 2.3 Synthesis of Ni/Ga and Ni/Fc

Ni/Ga and Ni/Fc were synthesised following the same hydrothermal procedure as for Ni, with the partial substitution of phthalic acid by Ga or Fc to introduce non-bridging co-ligands (Fig. 1).

For Ni/Ga, Ga (33.02 mg, 0.25 mmol) was added to the solvent mixture before nickel addition, maintaining a Ga : Ni^2+^ molar ratio of 1 : 6. The mixture was stirred, sealed, and heated at 140 °C for 48 h. The resulting product was collected by centrifugation (8000 rpm, 5 min), washed three times with ethanol, and dried in air.

For Ni/Fc, Fc (0.125 mmol) was added to the solvent mixture in the same manner, corresponding to a Fc : Ni^2+^ molar ratio of 1 : 12. The subsequent steps—hydrothermal reaction, centrifugation, washing, and drying—were identical to those used for Ni/Ga.

### 2.4 Material characterisation

The morphology and microstructure of the synthesised materials were analysed using field-emission scanning electron microscopy (FE-SEM, Hitachi S-4800, Japan) and transmission electron microscopy (TEM). Powder X-ray diffraction (XRD) patterns were obtained on a Bruker D8 Advance diffractometer (Germany) employing Cu Kα radiation (*λ* = 1.5406 Å) at 40 kV and 40 mA. Fourier-transform infrared (FT-IR) spectra were collected on a Nicolet iS50 spectrometer (Thermo Scientific, USA) in the range of 4000–400 cm^−1^ using KBr pellets.

Surface elemental composition and oxidation states were analysed *via* X-ray photoelectron spectroscopy (XPS, Thermo Scientific K-Alpha, USA) with Al Kα radiation. Specific surface areas and pore characteristics were determined by nitrogen adsorption–desorption isotherms at 77 K using a Micromeritics ASAP 2460 instrument. Brunauer–Emmett–Teller (BET) surface areas were calculated from the adsorption branch, and pore size distributions were derived using the Barrett–Joyner–Halenda (BJH) method.

### 2.5 Preparation of working electrodes

Catalyst inks were prepared by dispersing 5 mg of the as-prepared material (Ni, Ni/Ga, or Ni/Fc) in a mixed solvent containing 0.2 mL of ultrapure water, 0.3 mL of ethanol, and 20 µL of a 5 wt% Nafion solution. The suspension was ultrasonicated for 2 h to obtain a homogeneous ink. Subsequently, 15 µL of the ink was drop-cast onto a polished glassy carbon electrode (GCE, 3 mm diameter) and dried at room temperature under ambient air. The resulting electrodes were used directly for electrochemical measurements without any further treatment.

### 2.6 Electrochemical measurements

Electrochemical measurements were carried out on a CHI 760E electrochemical workstation (CH Instruments, China) using a standard three-electrode configuration at room temperature. A catalyst-coated glassy carbon electrode (GCE) served as the working electrode, a platinum wire as the counter electrode, and a saturated calomel electrode (SCE) as the reference electrode. Unless otherwise stated, potentials are reported *versus* SCE.

The electrolyte consisted of 1.0 mol L^−1^ KOH with or without 0.33 mol L^−1^ urea. Cyclic voltammetry (CV) was used to evaluate redox behaviour, while linear sweep voltammetry (LSV) was used to assess UOR activity. Electrochemical impedance spectroscopy (EIS) measurements were performed over a frequency range of 100 kHz to 0.1 Hz with a 5 mV amplitude to determine the charge-transfer resistance (*R*_ct_).

Chronoamperometry was carried out at a constant potential to assess catalyst stability. The electrochemically active surface area (ECSA) was estimated from the double-layer capacitance (*C*_dl_), derived from CV curves measured at various scan rates of 10–50 mV s^−1^ in the non-faradaic region.

## 3. Results and discussion

### 3.1 Structural, morphological, and surface characterisation

The three catalyst samples—Ni, Ni/Fc, and Ni/Ga—were synthesised *via* the one-step hydrothermal method described above and subjected to comprehensive structural, morphological, and surface characterisation. The incorporation of these non-bridging ligands was designed to introduce missing-linker defects, modify the Ni coordination environment, and influence the crystal morphology and porosity.

**Fig. 2:**
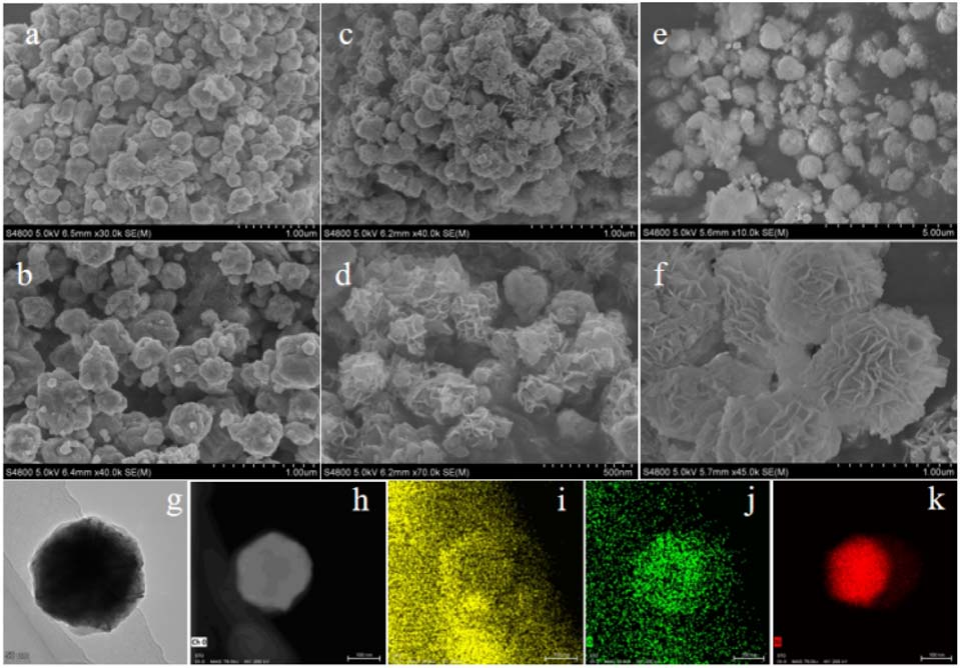
SEM images of (a and b) Ni, (c and d) Ni/Fc, and (e and f) Ni/Ga showing morphology evolution from spherical to lamellar architectures. (g and h) TEM image of Ni/Ga; (i–k) EDX elemental maps for C, O, and Ni, respectively corresponding to (h), confirming uniform elemental distribution. The lamellar and defect-enriched features in Ni/Ga indicate the effect of dual-ligand coordination on crystal growth and framework disorder.

SEM analysis (Fig. 2a–f) revealed that pristine Ni exhibited a uniform spherical morphology, whereas Ni/Fc formed cross-stacked lamellar structures, and Ni/Ga developed well-defined lamellar sheets surrounding the spheres. These morphological variations arise from ligand-induced modulation of the coordination framework, leading to altered nucleation and growth behaviour. TEM and EDX mapping (Fig. 2g–k) confirmed the uniform distribution of Ni, C, and O elements within the Ni/Ga framework, demonstrating successful incorporation of Ga ligands.

**Fig. 3:**
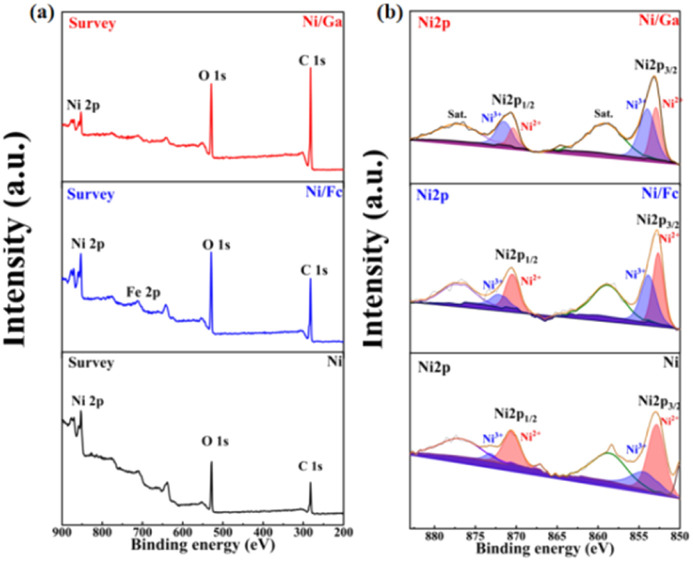
(a) XPS survey spectra of Ni, Ni/Fc, and Ni/Ga showing the presence of Ni, C, and O, with an additional Fe 2p signal in Ni/Fc. (b) High-resolution Ni 2p spectra displaying peaks corresponding to Ni^2+^ and Ni^3+^ species. The Ni^3+^/Ni^2+^ ratio increases in the order Ni < Ni/Fc < Ni/Ga, demonstrating that Ga incorporation promotes Ni oxidation and formation of catalytically active NiOOH.

X-ray photoelectron spectroscopy (XPS) was used to analyse the surface chemical states. Survey spectra (Fig. 3a) confirmed the presence of Ni, C, and O in all samples, with the additional appearance of an Fe 2p peak in Ni/Fc, indicating successful incorporation of Fc. High-resolution Ni 2p spectra (Fig. 3b) revealed the coexistence of Ni^2+^ and Ni^3+^ species in all samples, with peaks at ∼852.6 eV (Ni 2p_3/2_) and ∼870.1 eV (Ni 2p_1/2_). Notably, the Ni^3+^/Ni^2+^ ratio increased in the order: Ni < Ni/Fc < Ni/Ga. This suggests that the Ga-modified MOF framework facilitates oxidation of Ni^2+^, potentially creating more active NiOOH species, which are known to be key intermediates in the urea oxidation pathway.^24–26^

**Fig. 4:**
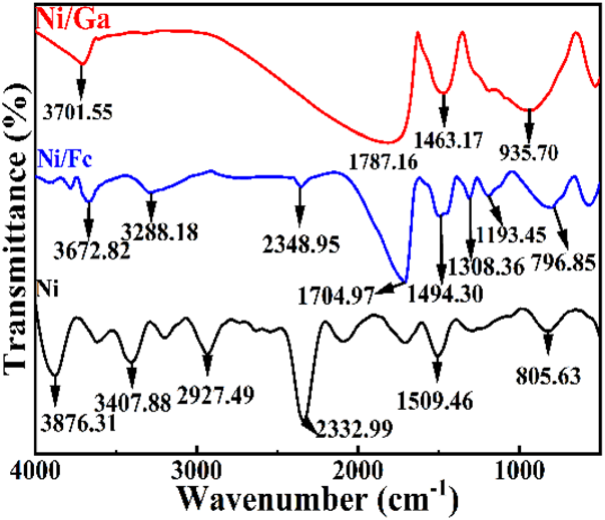
FT-IR spectra of Ni, Ni/Fc, and Ni/Ga in the 4000–400 cm^−1^ range. Broad O–H stretching bands and characteristic C=O vibrations (∼1705 cm^−1^) confirm surface hydroxyl and carbonyl functionalities. The intensity variation among samples reflects successful co-ligand incorporation and altered coordination environments.

FT-IR spectra (Fig. 4) displayed broad O–H stretching bands and strong C=O vibrations (∼1705 cm^−1^). The carbonyl band was more pronounced in Ni/Fc due to Fc's carbonyl groups, while Ni/Ga showed attenuated intensity, suggesting differences in coordination orientation. These results confirm successful ligand incorporation and variation in surface functional groups.

**Fig. 5:**
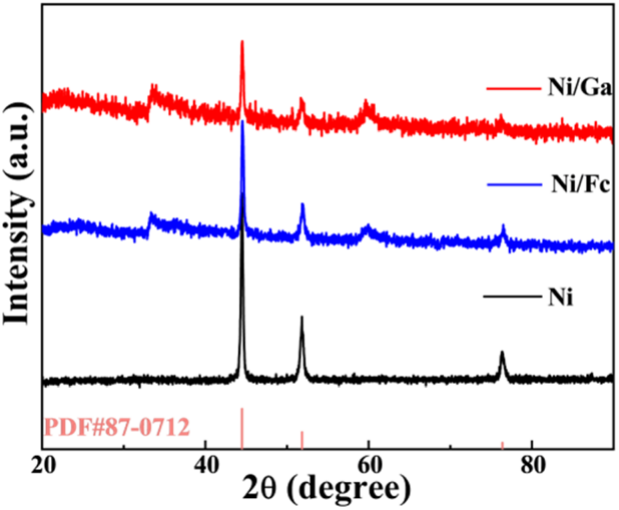
Powder X-ray diffraction (XRD) patterns of Ni, Ni/Fc, and Ni/Ga. All samples maintain the crystalline structure of nickel terephthalate hydrate, while slight peak broadening in Ni/Ga indicates partial amorphisation and defect formation induced by glutaric-acid incorporation.

Powder X-ray diffraction (XRD) patterns (Fig. 5) reveal that all samples retain their crystalline framework structure. The diffraction peaks of nickel show good agreement with the PDF #87-0712 reference pattern, with the peak around 44.5°, and those at 51.8° and 76.3° corresponding to the (111), (200), and (220) crystal planes of Ni, respectively. Although Fc and Ga doping did not significantly alter the primary crystal structure, the Ni/Ga samples exhibited slight peak broadening and impurity peaks (around 33° and 60°). This is consistent with the generation of surface defects and partial amorphisation resulting from Ga introduction.^27^

**Fig. 6:**
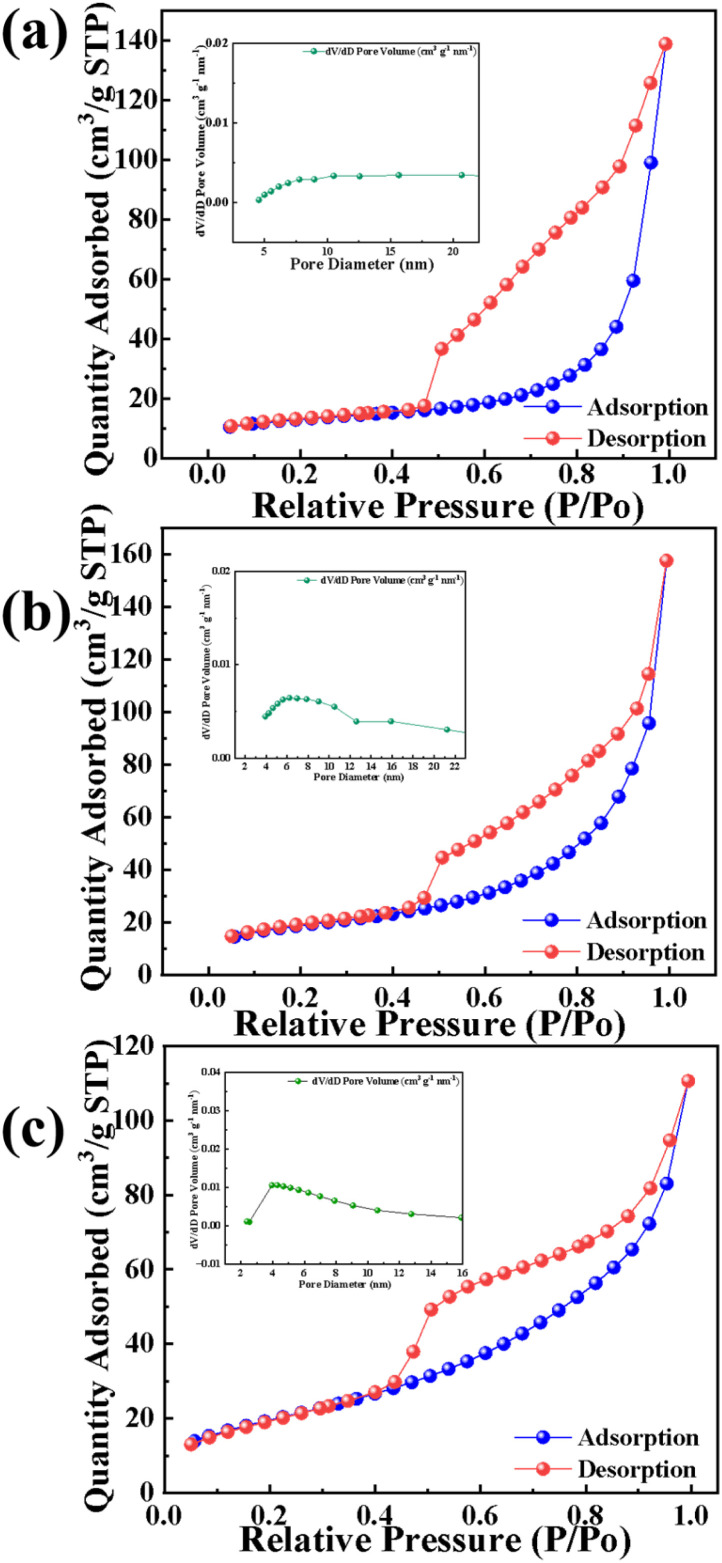
Nitrogen adsorption–desorption isotherms of (a) Ni, (b) Ni/Fc, and (c) Ni/Ga showing type III hysteresis loops characteristic of mesoporous materials. The inset compares BET surface areas: 47.21 m^2^ g^−1^ (Ni), 68.57 m^2^ g^−1^ (Ni/Fc), and 71.35 m^2^ g^−1^ (Ni/Ga). The enhanced surface area of Ni/Ga confirms glutaric-acid-induced defect formation and improved porosity, providing more accessible active sites for electrocatalysis.

The N_2_ adsorption–desorption isotherms (Fig. 6a) are classified as type III with H3-type hysteresis loops, indicating mesoporous structures with slit-shaped pores. Pore size distributions calculated from the adsorption branch *via* the BJH method (Fig. 6b) yield average pore diameters of 24.38 nm (Ni), 18.47 nm (Ni/Fc), and 12.57 nm (Ni/Ga). BET surface areas are 47.21 m^2^ g^−1^, 68.57 m^2^ g^−1^, and 71.35 m^2^ g^−1^ for Ni, Ni/Fc, and Ni/Ga, respectively. SEM images confirm similar spherical morphologies and particle sizes across all samples (Fig. 2), indicating that Fc and Ga modification do not alter the overall particle dimensions. Under this premise, the concurrent decrease in pore size and increase in surface area upon modification suggest the formation of a denser mesoporous network, particularly for Ni/Ga. This enhanced pore density provides more accessible active sites, contributing to its superior UOR performance.

**Fig. 7:**
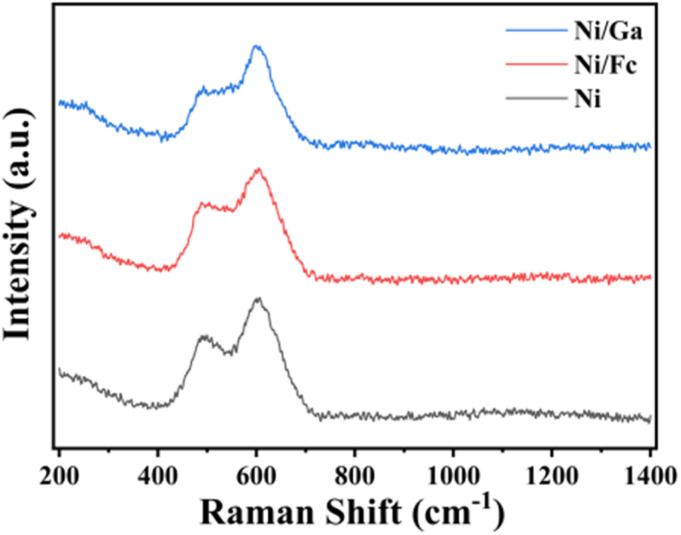
Raman spectra of Ni/Ga, Ni/Fc, and Ni samples.

The active species were characterised *via* Raman spectroscopy (Fig. 7). The spectra exhibited two distinct broad bands at approximately 480 cm^−1^ and 630 cm^−1^. The peak at 480 cm^−1^ is assigned to the characteristic bending vibration (E_g_ mode) of the Ni–O bond within the NiOOH species.^28^ Notably, the stretching vibration mode (A_1g_) of the Ni–O bond appears near 630 cm^−1^, exhibiting a significant blue shift compared to that of standard NiOOH (approximately 560 cm^−1^). This peak shift, accompanied by pronounced broadening, provides compelling evidence that the introduced Ga and Fc modifiers successfully induced a highly disordered surface structure with abundant lattice defects. Such distortion of the local coordination environment is favourable for the exposure of more highly reactive Ni^3+^ sites, thereby enhancing the kinetics of the urea oxidation reaction (UOR).

Notably, the pre-catalytic electronic and structural characteristics serve as critical indicators for subsequent UOR activity. The enrichment of surface high-valent Ni^3+^ species (confirmed by XPS spectra) and the disordered coordination environment (supported by the ∼630 cm^−1^ Raman peak) together establish a favourable pre-activated state. According to prior studies, such initial surface features can significantly facilitate rapid electrochemical reconstruction into the active NiOOH phase upon application of an anodic potential.^29–31^ In the present study, the superior performance of Ni/Ga stems from this optimised precursor state, which promotes the formation of a higher number of active sites and faster charge-transfer kinetics under steady-state catalytic conditions.^32^ It must be emphasised that the initial oxidation state and surface coordination environment of the catalyst precursor are pivotal factors determining its activation efficiency under operational conditions. Whilst XPS and Raman characterisation reflect the initial state, prior studies indicate that a higher proportion of Ni^3+^ in the precursor may serve as an “active centre seed”, promoting the electrochemical conversion to the active hydroxyl-oxidised nickel (NiOOH) phase under anodic potentials.^33–36^ In this study, the superior kinetic performance exhibited by Ni/Ga is attributed to ligand-induced charge transfer regulation. This regulatory mechanism pre-optimises the electron density at nickel sites, facilitating the stable maintenance of highly valent active species throughout the reaction process. Consequently, this accelerates the adsorption and oxidation of urea molecules.^37^

Collectively, these structural and compositional analyses confirm that dual-ligand coordination—particularly through glutaric acid—creates a defect-rich lamellar structure, enhances Ni^3+^ content, and increases surface area, all of which synergistically improve electrocatalytic activity for the urea oxidation reaction.^38–41^

### 3.2 Electrochemical evaluation of UOR activity

The urea oxidation reaction (UOR) performance of the three catalysts—Ni, Ni/Fc, and Ni/Ga—was assessed by cyclic voltammetry (CV) and linear sweep voltammetry (LSV) in 1.0 mol L^−1^ KOH, with or without 0.33 mol L^−1^ urea.

**Fig. 8:**
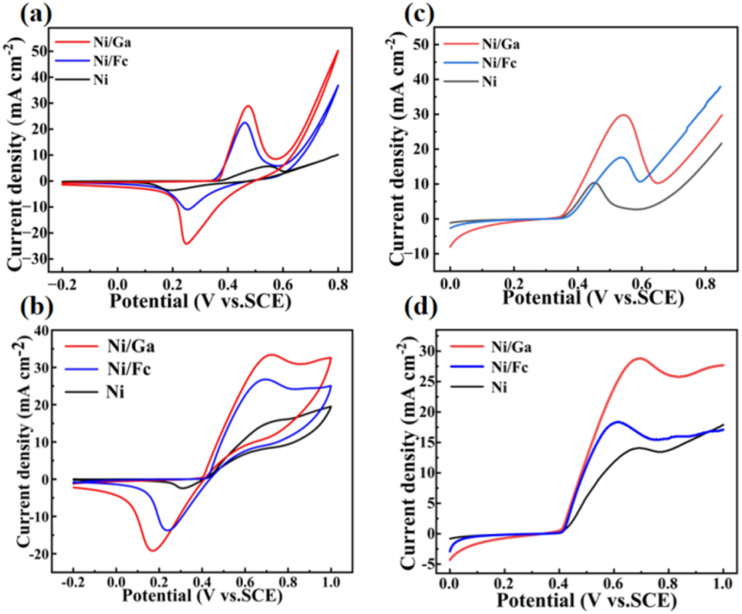
(a) CV curves of Ni, Ni/Fc, and Ni/Ga in 1.0 mol L^−1^ KOH showing Ni^2+^/Ni^3+^ redox transitions near 0.55 V. (b) CV curves in 1.0 mol L^−1^ KOH + 0.33 mol L^−1^ urea demonstrating enhanced catalytic current densities during urea oxidation. (c and d) LSV curves in KOH with and without urea. Ni/Ga exhibits the lowest onset potential (0.49 V *vs.* SCE) and highest current density, confirming superior UOR kinetics and Ni-site activation.

As shown in Fig. 8a, all catalysts exhibited a pair of Ni^2+^/Ni^3+^ redox peaks near 0.55 V, corresponding to the reversible formation of NiOOH species. The redox current increased upon introducing Fc and Ga ligands, suggesting enhanced redox reversibility and improved electrical conductivity. Notably, Ni/Ga displayed the broadest and most intense redox peaks, indicating superior Ni-site activation.

In the presence of urea (Fig. 8b), all materials exhibited a marked increase in current density, confirming UOR catalysis. The activity followed the order Ni/Ga > Ni/Fc > Ni, consistent with LSV results (Fig. 8c and d). The Ni/Ga electrode achieved a current density of 10 mA cm^−2^ at 0.49 V *vs.* SCE, which is lower than those of Ni/Fc (0.52 V) and Ni (0.56 V), demonstrating faster reaction kinetics and a lower energy barrier.

**Fig. 9:**
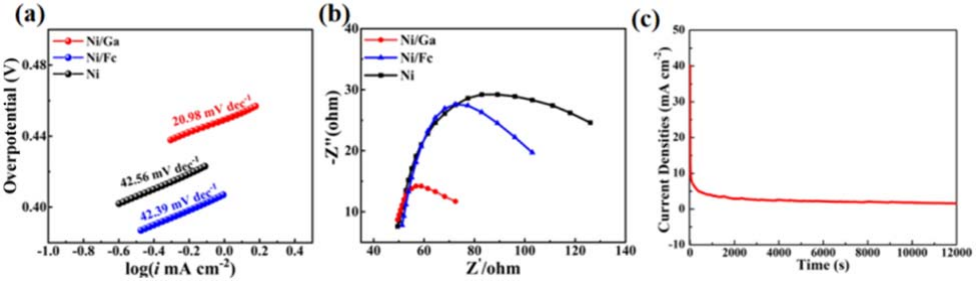
(a) Tafel plots of Ni, Ni/Fc, and Ni/Ga illustrating reaction kinetics. Ni/Ga shows the smallest Tafel slope (20.98 mV dec^−1^), indicating faster charge transfer. (b) Nyquist plots from EIS measurements in 1.0 mol L^−1^ KOH + 0.33 mol L^−1^ urea showing the lowest charge-transfer resistance (*R*_ct_) for Ni/Ga. (c) Chronoamperometric curve of Ni/Ga recorded at a constant potential demonstrating excellent operational stability and sustained catalytic activity.

Tafel analysis (Fig. 9a) revealed that Ni/Ga possesses the smallest Tafel slope (20.98 mV dec^−1^) among the three catalysts, markedly lower than those of Ni/Fc (42.39 mV dec^−1^) and Ni (42.56 mV dec^−1^), indicating significantly more favourable reaction kinetics. Consistently, EIS measurements (Fig. 9b) revealed the lowest charge-transfer resistance (*R*_ct_) for Ni/Ga, as evidenced by the smallest semicircle diameter in the Nyquist plot, confirming more efficient electron exchange pathways at the electrode–electrolyte interface.^42,43^ The concurrent observation of an ultralow Tafel slope and minimal *R*_ct_ provides mutually corroborating evidence that the tailored ligand coordination environment effectively promotes interfacial charge transfer, thereby accelerating UOR kinetics.

The chronoamperometric stability test (Fig. 9c) demonstrates that Ni/Ga maintains a stable current density (∼5 mA cm^−2^) after an initial transient, confirming good durability under continuous operation.

Collectively, these electrochemical results show that Ni/Ga exhibits the highest catalytic activity, fastest charge-transfer kinetics, and best operational stability among the three materials. The enhancements can be attributed to the higher Ni^3+^/Ni^2+^ ratio, defect-enriched lamellar morphology, and greater electroactive surface area generated by glutaric-acid-induced structural modulation.^44,45^

### 3.3 Electrochemically active surface area and stability

**Fig. 10:**
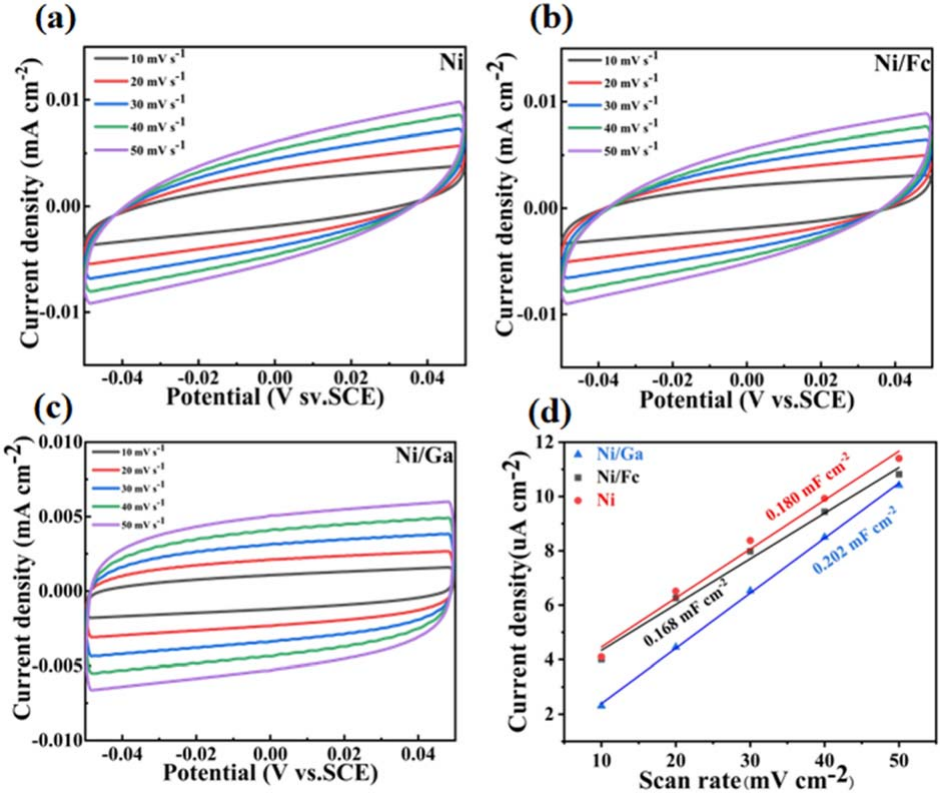
CV curves recorded at different scan rates (10–50 mV s^−1^) for (a) Ni, (b) Ni/Fc, and (c) Ni/Ga in the non-faradaic potential region. (d) Corresponding double-layer capacitance (*C*_dl_) calculations for Ni, Ni/Fc, and Ni/Ga. Ni/Ga exhibits the highest slope (*C*_dl_ = 0.202 mF cm^−2^), confirming its largest ECSA and the presence of more electrochemically accessible active sites arising from defect-rich lamellar morphology.

The electrochemically active surface area (ECSA) of the catalysts was estimated from the double-layer capacitance (*C*_dl_) by recording cyclic voltammetry (CV) curves at scan rates of 10–50 mV s^−1^ in the non-faradaic potential region (Fig. 10a–c). All three samples exhibited a linear relationship between current density and scan rate, confirming capacitive behaviour. Among them, Ni/Ga displayed the steepest slope, yielding a *C*_dl_ value of 0.202 mF cm^−2^, substantially higher than those of Ni/Fc and pristine Ni, indicating a significantly greater number of electrochemically accessible active sites.

The elevated *C*_dl_ and ECSA values of Ni/Ga are consistent with its higher BET surface area and defect-rich lamellar morphology identified in Section 3.1, collectively suggesting that glutaric-acid-induced surface structural disorder generates a denser network of accessible Ni active sites. These features facilitated more efficient urea adsorption and oxidation at the electrode surface, leading to improved catalytic kinetics.

The combination of high ECSA and sustained chronoamperometric stability collectively demonstrates that the Ni/Ga catalyst maintains both a large number of accessible active sites and structural integrity under continuous operational conditions. This correlation between surface structure and electrochemical activity further validated the ligand-directed defect engineering strategy, demonstrating that the introduction of glutaric acid effectively optimised the Ni coordination environment, exposed more active sites, and enhanced overall UOR performance,^46^ offering a rational and scalable design principle for durable nickel-based electrocatalysts.

The electrocatalytic performance of Ni/Ga is benchmarked against recently reported Ni-based catalysts (Table 1). Ni/Ga requires a low potential of 0.49 V (*vs.* SCE) to deliver 10 mA cm^−2^, surpassing many high-performance catalysts such as CoS_2_ (0.522 V). Most importantly, the Tafel slope of Ni/Ga (20.98 mV dec^−1^) is remarkably lower than those of the compared materials, signifying accelerated reaction kinetics. While stability is separately evidenced by *i*–*t* curves in Fig. 9c, the combined results confirm that the ligand-mediated surface engineering effectively optimises the active centres for superior urea oxidation.

**Table 1:** Comparison of onset potential at 10 mA cm^−2^ and Tafel slope for Ni/Ga against recently reported Ni-based UOR catalysts

Catalyst	*E* @ 10 mA cm^−2^ (V *vs.* SCE)	Tafel slope (mV dec^−1^)	Ref.
Ni/Ga	0.49	20.98	This work
Ni/MoC/Ti_3_C_2_T_*x*_@C	0.4926	84	47
4-Ni/CS	0.58	39	48
Ni(OH)_2_–graphene	0.362	—	49
Fe–Ni_3_S_2_@FeNi_3_-8	0.332	69	50
CoS_2_	0.522	80	51
Cu(OH)_2_/CuO	0.4316	—	52

## 4. Conclusions

In this work, a dual ligand-directed surface engineering strategy was developed to construct defect-rich nickel-based electrocatalysts, employing Ga and Fc as non-bridging surface modifiers alongside phthalic acid as the primary linker. Comparative characterisation of three systematically designed materials—Ni, Ni/Fc, and Ni/Ga—revealed that Ga modification imparted superior physicochemical properties, including a defect-rich mesoporous structure, an expanded electrochemically active surface area, and an optimised Ni^3+^/Ni^2+^ ratio. These structural attributes translated into outstanding UOR performance for Ni/Ga, evidenced by a low onset potential of 0.49 V (*vs.* SCE), an ultralow Tafel slope of 20.98 mV dec^−1^, and robust operational durability confirmed by chronoamperometric measurements. Mechanistic analysis demonstrated that Ga-induced surface structural defects play a pivotal role in modulating the coordination environment around Ni centres, accelerating interfacial charge transfer, and promoting the formation of the catalytically active NiOOH phase. Collectively, this work establishes a rational and scalable paradigm for ligand-directed defect engineering of nickel-based electrocatalysts, offering a practical design strategy for sustainable hydrogen production and the resource recovery of urea-rich wastewater.

## Author contributions

Wang Yifei: methodology, investigation, formal analysis, and writing – original draft. Li Jiayin: methodology, investigation, formal analysis, and writing – original draft. Luo An: investigation, software, writing – review and editing. Jin Yanxian: investigation, resources, formal analysis, writing – review and editing. Xu Wei: investigation, formal analysis, writing – review and editing. Chen Dan: investigation, formal analysis, writing – review and editing. Yu Hua: supervision, conceptualisation, investigation, and writing – review and editing. Worathat Thitikornpong: conceptualisation, investigation, formal analysis, and writing – review and editing. Yu Binbin: conceptualisation, methodology, investigation, formal analysis, supervision, resources, project administration, writing – review and editing.

## Conflicts of interest

The authors declare no conflicts of interest.

## Data Availability

The authors confirm that the data supporting the findings of this study are available within the article.
